# Negative end-expiratory versus zero end-expiratory pressure flow-controlled ventilation in a porcine hemorrhagic shock model

**DOI:** 10.1016/j.resplu.2025.101013

**Published:** 2025-06-21

**Authors:** Julia Abram, Patrick Spraider, Julian Wagner, Manuela Ranalter, Alexandra Gratl, Daniela Lobenwein, Sabine Wipper, Gabriel Putzer, Tobias Hell, Pia Tscholl, Judith Martini

**Affiliations:** a Department of Anesthesia and Intensive Care Medicine, Medical University Innsbruck, Anichstrasse 35, 6020 Innsbruck, Austria; b Department of Vascular Surgery, Medical University Innsbruck, Anichstrasse 35, 6020 Innsbruck, Austria; c Data Lab Hell, Non-University Research Institution, Europastrasse 2a, 6170 Zirl, Austria

**Keywords:** Negative end-expiratory pressure, Flow-controlled ventilation, Hemorrhagic Shock, Animal model

## Abstract

**Background:**

Hemorrhagic shock is a life-threatening event whereby low flow may lead to end-organ dysfunction. This is aggravated by mechanical ventilation, where a positive intrathoracic pressure further deteriorates venous filling of the heart. The aim of this study was to evaluate the effects of a mild negative end-expiratory pressure (NEEP) on mean arterial pressure compared to zero end-expiratory pressure (ZEEP) in a porcine hemorrhagic shock model.

**Methods:**

In anesthetized pigs, hemorrhagic shock was induced by a standardized lesion in the common femoral artery. After achieving half of baseline arterial pressure, hemorrhage was stopped. Ventilation was then switched to either flow-controlled ventilation with ZEEP (0 cmH_2_O, *n* = 6) or NEEP (−5 cmH_2_O, *n* = 6). After 5 min, fluid resuscitation was initiated (30 ml/kg) over 60 min and the observation period ended after 120 min.

**Results:**

The primary outcome parameter mean arterial pressure was significantly improved within the first 15 min with NEEP compared to ZEEP (49 vs 40 mmHg, MD 9 (95% CI 2 to 15); *p* = 0.031), but this effect diminished throughout fluid resuscitation. The cardiac index was similar in both groups. Evaluation of lung mechanics revealed a decrease in dynamic compliance (29 vs 44 ml/cmH_2_O, MD −14 (95% CI −20 to −9); *p* < 0.001) in the NEEP group without differences in gas exchange.

**Conclusions:**

Application of −5 cmH_2_O NEEP improved mean arterial pressure but not cardiac index during hemorrhagic shock and the first 15 min of fluid resuscitation compared to ZEEP. Thereby gas exchange performance was similar.

## Highlights

• Hemodynamics during hemorrhagic shock are impaired by positive pressure ventilation.
• Negative compared to zero end-expiratory pressure improves mean arterial pressure.
• This only applies to the hypovolemic state and diminishes during fluid resuscitation.
• Gas exchange is thereby not significantly impaired.

## Background

Hemorrhagic shock may occur due to traumatic injury, maternal- and gastrointestinal hemorrhage, rupture of an aneurysm, or perioperatively. Regardless of its cause, a hypovolemic condition reduces stroke volume of the heart, lowers arterial blood pressure and consecutively reduces overall oxygen delivery.^1^ This condition is further aggravated if artificial ventilation is necessary and positive intrathoracic pressure of mechanical ventilation deteriorates venous return to the heart. Because of this well-known effect^2^, end-expiratory pressure is frequently set to zero, to lower intrathoracic pressure and thus improve hemodynamic conditions.^3^ Reducing end-expiratory pressure, however, adversely impacts lung function, necessitating a careful balance between these negative effects and the potential benefits associated with enhanced venous return. A further decrease of end-expiratory pressure to negative values has been shown to improve mean arterial blood pressure and cardiac output.^4^ In this study, however, excessive negative pressures of up to −30 cmH_2_O were used, which is very likely to worsen lung function and increase the risk of atelectasis.

Flow-controlled ventilation is an emerging ventilation mode that is unique in controlling expiratory gas flow. Besides the constant gas flow during the entire ventilation cycle, the active control of expiration allows for any end-expiratory pressure setting, even in the negative range. This easily applicable setting might be beneficial in hemorrhagic shock and represents the rationale for performing this study.

The aim of this trial was to investigate whether a negative end-expiratory pressure (NEEP) of −5 cmH_2_O will improve the primary outcome parameter mean arterial pressure during hemorrhagic shock compared to zero end-expiratory pressure (ZEEP). Additional outcome parameters were cardiac output, respiratory parameters such as compliance, resistance, and gas exchange parameters (PaO_2_ and PaCO_2_).

## Methods

### Animal preparation

Experiments were performed on 12 domestic pigs of either sex (50% female) and a body weight of 70 to 100 kg. Animals were fasted overnight with free access to water. Premedication was performed with intramuscular injection of azaperone (4 mg/kg) and atropine (0.01 mg/kg) one hour before transportation to the experimental facility. Deeper sedation was induced by an intramuscular injection of ketamine (30 mg/kg). Subsequently, an ear vein was cannulated and orotracheal intubation was performed during spontaneous breathing (tracheal tube with an internal diameter of 8.0 mm; Willy Rüsch GmbH, Kernen, Germany). Anesthesia was induced through a single bolus dose of propofol (2 mg/kg) and rocuronium (1 mg/kg) and maintained through a continuous infusion of propofol (6–8 mg/kg/h), remifentanil (0.2–0.3 µg/kg/min) and rocuronium (0.5 mg/kg/h). Normovolemia was maintained by infusion of balanced crystalloid solution (5–10 ml/kg/h Elomel iso®; Fresenius Kabi Austria GmbH, Graz, Austria). To prevent septic complications 1.5 g cefuroxime was administered and repeated after four hours. This regime has been proven to guarantee an appropriate depth of anesthesia without hemodynamic disturbances.^6^, ^7^, ^8^

For baseline ventilation volume-controlled ventilation (VCV) was performed (EvitaXL®, Dräger Medical, Lübeck, Germany) with FiO_2_ set at 0.3 and a tidal volume (V_T_) of 8 ml/kg body weight. PEEP was set to 5 cmH_2_O and inspiration to expiration ratio (I:E) to 1:1.5. Breathing frequency was adjusted to maintain normocapnia (PaCO_2_ 35 to 45 mmHg).

A Leader-Cath arterial catheter (4F; Vygon, Swindon, UK) was placed into the carotid artery for invasive arterial pressure monitoring and arterial blood gas sampling. A pulmonary artery catheter (7F; Edwards Life Science, Irvine, CA, USA) was placed into the right pulmonary artery via the right internal jugular vein after ultrasound-guided introducer sheath insertion (8.5F; Arrow, Reading, PA, USA). A pig-tail catheter (8F; Navarre® Opti-Drain®, Bard, Tempe, USA) was placed into the bladder after ultrasound-guided puncture for urine release. The left femoral artery was surgically exposed.

After obtaining baseline measurement parameters, a standardized lesion to the left common femoral artery using a 4.7 mm puncher (aortic puncher, Aesculap® Surgical Instruments, Braun, Melsungen, Hessen, Germany) was performed. Hemorrhage was maintained until the initial systolic pressure was halved and then stopped with the application of a hemostyptic agent (QuikClot® Combat Gauze, Z-Medica, Wellingford, CT, USA or Polyphosphat, Speed Care Mineral, Neubrandenburg, Germany) and standardized compression using a FemoStop® device (Abbott Medical, Plymouth, Illionis, USA) with 200 mmHg pressure. The hemostatic intervention was balanced between the NEEP and ZEEP group (both 3/6, 50%). Subsequently, hemorrhagic shock measurement parameters were recorded.

### Experimental protocol

After animal preparation, baseline- and hemorrhagic shock measurements were obtained during VCV ventilation. Subsequently, ventilation was switched to flow-controlled ventilation (FCV) (Evone®, Ventinova Medical B.V., Eindhoven, the Netherlands) and animals were randomly allocated (to receive ZEEP, with 0 cmH_2_O or NEEP, with −5 cmH_2_O) based on a randomization list generated using the random permuted blocks method with varying block size (Stata version 17.0, Ralloc version 3.7.6). Otherwise, peak pressure was adjusted to achieve a tidal volume (V_T_) of 8 ml/kg and the gas flow was set to achieve normocapnia in both groups. After five minutes, the FemoStop® device was removed, local hemostasis was checked by the surgeon and fluid resuscitation was initiated and continuously carried forward over a period of 60 min with balanced crystalloid solution (30 ml/kg Elomel iso®; Fresenius Kabi Austria GmbH, Graz, Austria). After 60 min, fluid resuscitation was stopped, and the animals were observed for additional 60 min without any further manipulation of the animals.

### Measurement variables and timepoints

Recorded and analyzed respiratory parameters included peak pressure (P_peak_), driving pressure (ΔP), V_T_, respiratory rate (RR), respiratory minute volume (MV), dynamic resistance (R), and compliance (C). Cardiovascular monitoring included the primary outcome parameter mean arterial pressure (MAP), heart rate (HR), and mean pulmonary artery pressure (MPAP). Cardiac output, systemic- and pulmonary vascular resistance were measured via pulmonary artery catheter after three consecutive injections of 10 ml of saline. The index of these parameters was calculated using the predicted body surface area for pigs.^9^ Metabolic parameters were assessed based on arterial and mixed-venous blood gas samples where pH, arterial partial pressure of CO_2_ (PaCO_2_), O_2_ (PaO_2_), and mixed-venous oxygen saturation (SvO_2_) were measured (ABL800 Flex®; Radiometer, Copenhagen, Denmark).

After obtaining baseline measurements before (BL) and after hemorrhagic shock induction (S), further measurement timepoints were defined after randomization to ZEEP or NEEP at 5, 10, and 15 min after shock induction to evaluate prompt consequences of different end-expiratory pressure settings before and after start of fluid resuscitation. Subsequently, measurement timepoints were defined at 30, 60, 90, and 120 min after shock induction.

### Statistical analysis

This study was performed as a supplement of a vascular surgery trial investigating two different hemostatic agents. Therefore, the sample size was set at 12 animals, which is appropriate for the investigation of an unknown effect with high clinical relevance.

The statistical analysis was performed using R, version 4.0.3 (The R Foundation, Vienna, Austria). For the characteristics of laboratory animals before the start of the experiment, continuous data are presented as median (25th to 75th percentile) and categorical variables as frequencies (%). Effect size and precision are shown with estimated median differences between groups for continuous data and odds ratios for binary variables with 95% confidence intervals (CI). The Wilcoxon rank sum test and Fisher's exact test were applied to assess group differences.

The course of hemodynamic parameters during the first 15 min (5, 10, and 15 min after hemorrhagic shock) and the entire observation period (over 120 min) are shown per group using the median course with corresponding 95% CI’s. Differences between groups were assessed using linear mixed-effects models with *group* as fixed effect as well as *time* and *subjects* as random intercepts. To assess the statistical power of the fitted linear mixed-effects model comparing NEEP and ZEEP groups (time window: 5–15 min) with respect to cardiac index, a post hoc power analysis was performed using the R package simr and its powerSim function. All statistical assessments were two-sided, a significance level of 5% was used.

## Results

After enrollment of 12 animals (ZEEP *n* = 6; NEEP *n* = 6), all pigs were included in the primary analysis. Demographics and characteristics at baseline and after hemorrhagic shock before randomization were comparable between groups (Table 1).

**Table 1:** Demographic data and baseline characteristics of animals.

	**Total**^a^ **(*n* = 12)**	**NEEP**^a^ **(*n* = 6)**	**ZEEP**^a^ **(*n* = 6)**	**estimate with 95% CI**^b^	**p-value**^c^
demographic data					
weight	86 (74–94)	76 (73–85)	95 (87–96)	−16 (−27 to 3)	0.092
sex [female]	6/12 (50%)	3/6 (50%)	3/6 (50%)		1
blood loss [ml]	1803 (1683–2016)	1719 (1671–1722)	1936 (1885–2043)	−217 (−1277 to 367)	0.421

**hemodynamic parameters**					
HR [/min]					
baseline	62 (58–84)	76 (60–95)	58 (57–64)	9 (0 to 41)	0.075
shock	98 (78–126)	110 (85–130)	91 (75–111)	14 (−31 to 57)	0.394
MAP [mmHg]					
baseline	79 (74–85)	86 (77–88)	77 (72–80)	7 (−8 to 19)	0.240
shock	31 (28–32)	31 (29–33)	29 (28–31)	3 (−3 to 6)	0.324
CVP [mmHg]					
baseline	11 (9–12)	11 (813)	11 (9–26)	0 (−4 to 4)	1
shock	6 (5–7)	6 (6–7)	6 (4–8)	0 (−3 to 2)	0.870
MPAP [mmHg]					
baseline	25 (20–28)	26 (22–29)	23 (19–26)	3 (−7 to 11)	0.470
shock	13 (12–14)	14 (12–15)	13 (11–13)	1 (−5 to 5)	0.628
PCWP [mmHg]					
baseline	12 (9–14)	11 (9–12)	14 (12–17)	−3 (−8 to 2)	0169
shock	6 (4–8)	6 (4–7)	7 (5–8)	−1 (−5 to 2)	0.416
CI [l/min/m^2^]					
baseline	5.3 (4.5–7.1)	6.2 (4.7–8.1)	5.1 (4.5–5.7)	1.0 (−1.3 to 4.0)	0.485
shock	2.2 (1.9–2.8)	2.3 (2.1–3.0)	2.0 (1.8–2.6)	0.3 (−0.6 to 1.4)	0.416
PVRI [dyn·s/cm^5^/m^2^]					
baseline	93 (73–120)	115 (85–159)	76 (65–109)	33 (−18 to 102)	0.132
shock	164 (110–184)	181 (145–196)	113 (108–164)	44 (−27 to 161)	0.178
SVRI [dyn·s/cm^5^/m^2^]					
baseline	475 (398–758)	551 (379–839)	475 (452–500)	−49 (−136 to 471)	0699
shock	486 (420–610)	549 (480–630)	437 (382–499)	96 (−49 to 240)	0.310

**respiratory parameters**					
respiratory rate [/min]					
baseline	24 (24–26)	26 (24–27)	24 (21–24)	3 (−1 to 7)	0.157
shock	24 (23–25)	25 (24–27)	24 (21–25)	2 (−1 to 6)	0.324
tidal volume [ml/kg]					
baseline	8.0 (7.9–8.1)	7.9 (7.8–8.0)	8.1 (8.0–8.1)	−0.1 (−0.4 to 0.2)	0.240
shock	8.0 (7.8–8.1)	7.9 (7.7–8.1)	8.0 (8.0–8.1)	−0.1 (−0.5 to 0.2)	0.485
minute volume [l/min]					
baseline	16.1 (14.6–17.9)	15.8 (14.9–17.1)	16.7 (14.2–18.0)	−0.3 (−3.1 to 4)	1
shock	15.9 (13.7–18.0)	15.9 (14.1–16.8)	16.4 (14.1–18.7)	−0.1 (−4.0 to 4.8)	1
peak pressure [cmH_2_O]					
baseline	21 (20–23)	20 (19–22)	21 (20–23)	−1 (−4 to 3)	0.685
shock	21 (20–23)	21 (19–23)	22 (21–23)	−1 (−3 to 2)	0.626
driving pressure [cmH_2_O]					
baseline	16 (15–18)	15 (14–17)	16 (15–18)	−1 (−4 to 3)	0.685
shock	16 (15–18)	16 (14–18)	17 (16–18)	−1 (−3 to 2)	0.626
compliance [ml/cmH_2_O ]					
baseline	61 (56–63)	59 (53–61)	63 (58–67)	−5 (−21 to 4)	0.240
shock	59 (53–65)	57 (52–59)	63 (55–69)	−6 (−19 to 8)	0.310
resistance [cmH_2_O·s/l]					
baseline	8.8 (8.2–9.5)	9.3 (8.6–10.1)	8.2 (8.0–8.9)	1.1 (−0.1 to 2.4)	0.093
shock	9.0 (8.5–9.8)	9.4 (9.0–10.2)	8.6 (8.3–9.4)	0.8 (−0.6 to 2.9)	0.172

**metabolic parameters**					
pH					
baseline	7.44 (7.42–7.49)	7.43 (7.41–7.45)	7.47 (7.43–7.49)	−0.03 (−0.09 to 0.07)	0.394
shock	7.46 (7.44–7.49)	7.47 (7.46–7.48)	7.45 (7.44–7.49)	0.02 (−0.08 to 0.08)	0.699
PaCO_2_ [mmHg]					
baseline	41.2 (40.4–43.5)	41.2 (40.9–43.6)	41.2 (40.2–43.0)	0.7 (−4.9 to 5.7)	0.699
shock	36.2 (33.9–38.2)	34.5 (33.1–37.0)	36.6 (36.1–40.0)	−2.5 (−7.7 to 2.3)	0.310
PaO_2_ [mmHg]					
baseline	127 (117–131)	124 (110–141)	127 (125–128)	1 (−24 to 32)	1
shock	325 (308–352)	332 (293–362)	325 (317–336)	4 (−49 to 72)	1
Hb [mg/dl]					
baseline	10.1 (9.3–10.4)	10.4 (9.9–10.7)	9.5 (8.5–10.2)	0.9 (−0.2 to 2.3)	0.128
shock	9.4 (8.5–9.8)	9.8 (9.0–10.2)	8.7 (8.1–9.5)	0.8 (−0.3 to 2.2)	0.109
lactate [mg/dl]					
baseline	18 (15–25)	25 (18–28)	15 (13–18)	9 (−1 to 31)	0.065
shock	28 (22–33)	31 (35–45)	28 (20–28)	6 (−6 to 21)	0.227
SvO_2_ [%]					
baseline	65 (57–74)	72 (61–74)	62 (56–66)	7 (–22 to 19)	0.485
shock	38 (35–43)	38 (35–45)	38 (25–43)	10 (−8 to 22)	0.537

CI = cardiac index, CVP = central venous pressure, Hb = hemoglobin concentration, HR = heart rate, MAP = mean arterial pressure, MPAP = mean pulmonary arterial pressure, MV = respiratory minute volume, NEEP = negative end-expiratory pressure, PaCO_2_ = arterial partial pressure of carbon dioxide, PaO_2_ = arterial partial pressure of oxygen, PCWP = pulmonary capillary wedge pressure, PVRI = pulmonary vascular resistance index, SVRI = systemic vascular resistance index, SvO_2_ = mixed-venous oxygen saturation, ZEEP = zero end-expiratory pressure;

a Binary data are presented as no./total no. (%), continuous data as medians (25th to 75th percentile).

b Estimated median difference for continuous variables.

c Assessed by Fisher’s exact test for categorical variables and Wilcoxon rank sum test for continuous variables, significant differences (*p* < 0.05) are marked with an asterisk.

Evaluation of the first 15 min (timepoint 5, 10, and 15 min) after switching to ZEEP or NEEP revealed a significantly higher MAP in NEEP (49 vs 40 mmHg, MD 9 (95% CI 2 to 15); *p* = 0.031) compared to ZEEP animals. All other hemodynamic parameters were comparable between groups (Table 2). Results of the respiratory parameters revealed a higher driving pressure (19 vs 16 cmH_2_O, MD 3 (95% CI 1 to 5); *p* = 0.022) and a lower dynamic compliance (32 vs 45 ml/cmH_2_O, MD −13 (95% CI −19 to −8); *p* < 0.001) when using NEEP instead of ZEEP. Metabolic analysis of blood gas samples showed similar gas exchange parameters (PaO_2_ and PaCO_2_, Table 3), but a higher SvO_2_ was observed in NEEP (56 vs 42%, MD 14 (95% CI 4 to 25); *p* = 0.027).

Analysis of the entire study period (0 to 120 min) revealed no significant difference in MAP between groups (Table 2). Only the hemodynamic parameter PCWP differed and was lower in NEEP compared to ZEEP (5 vs 9 mmHg, MD −4 (95% CI −7 to −1); *p* = 0.033). Evaluation of the respiratory variables revealed a higher driving pressure (19 vs 16 cmH_2_O, MD 3 (95% CI 1 to 5); *p* = 0.022), a lower dynamic compliance (29 vs 44 ml/cmH_2_O, MD −14 (95% CI −20 to −9); <0.001) and resistance (6.9 vs 5.3 cmH_2_O·s/l, MD 1.6 (95% CI 0.6 to 2.5); *p* = 0.008) for the NEEP group. All metabolic parameters were comparable between groups (Table 3).

**Table 2:** Course of hemodynamic parameters during hemorrhagic shock with estimated differences between groups.

		**NEEP**^a^ **(*n* = 6)**	**ZEEP**^a^ **(*n* = 6)**	**estimate with** **95% CI**^b^	**p-** **value**^c^
HR [/min]					
	0–15 min	80 (63–97)	71 (49–93)	9 (−13 to 31)	0.428
	0–120 min	81 (68–94)	74 (56–91)	7 (−10 to 25)	0.426
MAP [mmHg]					
	0–15 min	49 (40–58)	40 (34–47)	9 (2 to 15)	0.031*
	0–120 min	64 (51–76)	56 (47–64)	8 (0 to 16)	0.093
MPAP [mmHg]					
	0–15 min	12 (9–15)	14 (10–18)	−2 (−6 to 2)	0.321
	0–120 min	16 (12–19)	18 (14–21)	−2 (−6 to 1)	0.251
CVP [mmHg]					
	0–15 min	5 (3–6)	6 (4–8)	−1 (−3 to 0)	0.168
	0–120 min	6 (4–7)	6 (4–8)	−1 (−3 to 1)	0.383
PCWP [mmHg]					
	0–15 min	3 (1–5)	6 (3–9)	−3 (−6 to 0)	0.051
	0–120 min	5 (2–7)	9 (6–12)	−4 (−7 to −1)	0.033*
CI [l/min/m^2^]					
	0–15 min	4.4 (3.1–5.7)	3.4 (2.2–4.7)	1.0 (−0.3 to 2.2)	0.162
	0–120 min	5.7 (4.2–7.3)	4.9 (3.4–6.4)	0.8 (−0.7 to 2.3)	0.311
SVRI [dyn·s/cm^5^/m^2^]					
	0–15 min	537 (421–653)	476 (312–641)	61 (−104 to 225)	0.486
	0–120 min	531 (410–653)	472 (301–644)	59 (−113 to 231)	0.517
PVRI [dyn·s/cm^5^/m^2^]					
	0–15 min	114 (92–135)	100 (71–129)	14 (−16 to 43)	0.383
	0–120 min	101 (81–120)	83 (59–106)	18 (−6 to 42)	0.165

CI = cardiac index, CVP = central venous pressure, HR = heart rate, MAP = mean arterial pressure, MPAP = mean pulmonary arterial pressure, NEEP = negative end-expiratory pressure, PCWP = pulmonary capillary wedge pressure, PVRI = pulmonary vascular resistance index, SVRI = systemic vascular resistance index, ZEEP = zero end-expiratory pressure;

a Continuous data are presented as medians with 95% CI.

b Estimated median difference for continuous variables.

c Assessed by Wilcoxon rank sum test for continuous variables, significant differences (*p* < 0.05) are marked with an asterisk.

**Table 3:** Course of respiratory and metabolic parameters during hemorrhagic shock with estimated differences between groups.

		**NEEP**^a^ **(*n* = 6)**	**ZEEP**^a^ **(*n* = 6)**	**estimate with** **95% CI**^b^	**p-** **value**^c^
tidal volume [ml/kg]					
	0–15 min	8.0 (7.9–8.1)	8.1 (7.9–8.2)	0.0 (−0.2 to 0.1)	0.558
	0–120 min	8.0 (7.9–8.1)	8.1 (8.0–8.2)	−0.1 (−0.2 to 0)	0.279
respiratory rate [/min]					
	0–15 min	14 (12–15)	12 (10–14)	2 (0 to 4)	0.109
	0–120 min	14 (12–15)	12 (10–14)	2 (0 to 4)	0.148
minute volume [l/min]					
	0–15 min	8.6 (8.4–8.8)	8.6 (8.3–8.9)	0.0 (−-0.3 to 0.3)	0.834
	0–120 min	8.6 (8.4–8.8)	8.7 (8.4–9.0)	−0.1 (−0.4 to 0.1)	0.356
peak pressure [cmH_2_O]					
	0–15 min	14 (12–15)	16 (14–18)	−2 (−4 to 0)	0.065
	0–120 min	15 (13–16)	16 (14–18)	−2 (−3 to 0)	0.157
driving pressure [cmH_2_O]					
	0–15 min	19 (17–20)	16 (14–18)	3 (1 to 5)	0.022*
	0–120 min	20 (18–21)	16 (14–18)	3 (2 to 5)	0.005*
compliance [ml/cmH_2_O]					
	0–15 min	32 (28–35)	45 (39–50)	−13 (−19 to −8)	<0.001*
	0–120 min	29 (25–34)	44 (38–49)	−14 (−20 to −9)	<0.001*
resistance [cmH_2_O·s/l]					
	0–15 min	6.6 (5.6–7.6)	5.1 (3.8–6.4)	1.5 (0.2 to 2.9)	0.051
	0–120 min	6.9 (6.2–7.6)	5.3 (4.4–6.3)	1.6 (0.6 to 2.5)	0.008*
PaO_2_ [mmHg]					
	0–15 min	304 (266–342)	322 (273–372)	−18 (−68 to 31)	0.488
	0–120 min	288 (254–323)	305 (261–349)	−17 (−61 to 27)	0.471
PaCO_2_ [mmHg]					
	0–15 min	45.5 (39.8–51.3)	45.3 (37.8–52.8)	0.2 (−7.3 to 7.7)	0.953
	0–120 min	47.4 (40.5–54.3)	49.0 (39.7–58.4)	−1.6 (−11.0 to 7.8)	0.742
Hb [mg/dl]					
	0–15 min	8.8 (8.0–9.6)	8.2 (7.2–9.2)	0.6 (−0.3 to 1.6)	0.227
	0–120 min	8.0 (7.2–8.8)	7.6 (6.8–8.5)	0.3 (−0.5 to 1.2)	0.437
lactate [mg/dl]					
	0–15 min	33 (24–41)	29 (17–42)	3 (−9 to 16)	0.609
	0–120 min	27 (18–35)	26 (16–36)	1 (−9 to 10)	0.902
SvO_2_ [%]					
	0–15 min	56 (45–67)	42 (31–52)	15 (4–25)	0.027*
	0–120 min	63 (53–74)	52 (41–63)	11 (0 to 22)	0.076

Hb = hemoglobin concentration; PaCO_2_ = arterial partial pressure of carbon dioxide, PaO_2_ = arterial partial pressure of oxygen, SvO_2_ = mixed-venous oxygen saturation;

a Continuous data presented as medians with 95% CI.

b Estimated median difference for continuous variables.

c Assessed by Wilcoxon rank sum test for continuous variables, significant differences (*p* < 0.05) are marked with an asterisk.

## Discussion

**Fig. 1:**
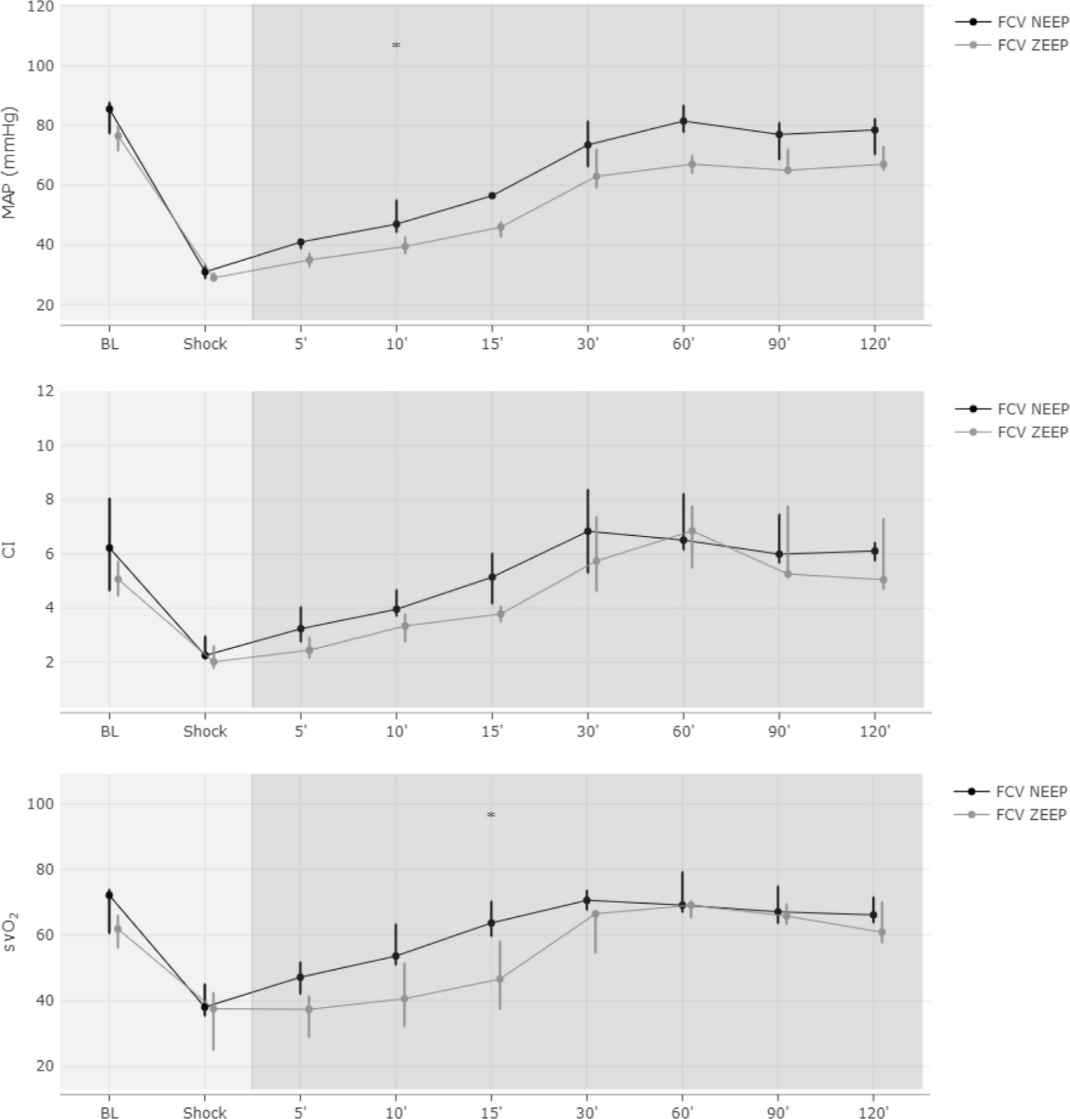
Demonstrates the median (interquartile range are displayed as deviance) course of arterial pressure (MAP, mmHg), cardiac index (CI, l/min/m^2^) and mixed-venous oxygen saturation (SvO_2_) after baseline measurement (BL) and induction of hemorrhagic shock in 12 animals treated with either flow-controlled ventilation and zero end-expiratory pressure (FCV ZEEP, grey line) or FCV and a negative end-expiratory pressure of -5 cmH_2_O (FCV NEEP, black line). After 5 min, fluid resuscitation was administered continuously over a periof of 60 min.

In this experimental model of hemorrhagic shock, ventilation with negative end-expiratory pressure significantly increased mean arterial pressure compared to zero end-expiratory pressure; this effect was observed during the initial 15 min of fluid resuscitation. However, this hemodynamic improvement diminished as fluid resuscitation progressed (Fig. 1). It can therefore be concluded, as demonstrated in previous studies^4^, ^10^, that negative end-expiratory pressure may enhance venous return to the heart and subsequently increase mean arterial pressure (MAP), though this effect appears to be limited to the hypovolemic state. Surprisingly, this assumption is not supported by cardiac index values, which did not statistically differ between groups. However, there is a trend in increased cardiac index for NEEP animals (Fig. 1), which might be clinically meaningful (4.4 vs 3.4 l/min/m^2^, corresponding to a 22% increase in CI with NEEP compared to ZEEP); due to the low number of animals, the variance is too high and the trial underpowered to draw any statistically significant conclusions (achieved power of 0.36, 95% CI 0.27–0.46).

Another factor that supports the assumption that NEEP is capable of improving hemodynamic stability during hemorrhagic shock is the finding that SvO_2_ was significantly higher in NEEP animals compared to ZEEP. Because the oxygen content (PaO_2_ and Hb) was comparable between groups, improved oxygen delivery leading to a higher SvO_2_ can be assumed. Increased oxygen extraction as an alternative explanation seems rather unlikely, as the difference in SvO_2_ was only observable during the hypovolemic state and not before or after. In terms of lactate concentration, we observed a difference at baseline that was not significant but nevertheless noteworthy, which we are unable to explain. This difference evened out by the time of hemorrhagic shock and followed a similar course for the remainder of the study. We therefore assume that this was a random observation with no significant impact on the study.

Our results are in line with a previous experimental trial investigating NEEP up to −30 cmH_2_O^4^ where an even more pronounced effect on MAP and CI was found. Similarly, Berlin et al. found an increase in MAP, CI and stroke volume when using an expiratory ventilation assistance (EVA) and a NEEP of −8 cmH_2_O.^10^

In contrast to the positive influence on hemodynamics, a deterioration in lung function was expected due to the application of NEEP and the accompanying increased risk of atelectasis. Indeed, NEEP animals required higher driving pressures, although transmission of negative pressures to the lung periphery cannot be assumed with certainty. Dynamic compliance measured independently of the driving pressure revealed a decrease in dynamic lung compliance indicating deterioration of lung mechanics. Oxygenation and the required minute volume for CO_2_ removal showed no significant differences between the groups, even after two hours. Thus, the short-term application of NEEP appears to be well-tolerated without compromising gas exchange. In the critical context of acute hemorrhage accompanied by severe hemodynamic instability, NEEP seems to be a viable short-term strategy for patient stabilization.

Our study has several limitations. First, the results of a porcine model cannot be directly transferred to humans because of anatomical and metabolic differences. Quadrupeds have a significantly lower chest wall compliance, different lung mechanics and a significantly increased metabolism. This leads to an enhanced susceptibility to hypovolemia and impaired hemodynamic stability. Second, we solely investigated a NEEP of −5 cmH_2_O and found mild effects on hemodynamic parameters. A further decrease in end-expiratory pressure may enhance the observed beneficial effects. Third, we did not investigate the presence of atelectasis and can therefore only assume that the change in lung mechanics is due to de-recruitment of lung tissue. Furthermore, we did not examine whether the potential negative effects on lung mechanics could be mitigated by applying a higher PEEP level or performing a recruitment maneuver following successful fluid resuscitation and the restoration of normovolemia. Finally, this study was conducted to supplement to a vascular surgery study. Although both the hemostatic agents investigated were sufficiently effective in stopping femoral bleeding, bias and confounding errors cannot be ruled out.

One advantage of this study is the easy applicability of the investigated method of NEEP in hemorrhagic shock in the clinical setting. For this purpose, flow-controlled ventilation was used with a commercially available ventilator that is certificated and increasingly used in Europe. In case of a bleeding event, a NEEP can be applied at any time simply by negating the set end-expiratory pressure.

## Conclusion

In this experimental study, flow-controlled ventilation with a negative end-expiratory pressure of −5 cmH_2_O improved mean arterial pressure during hemorrhagic shock and the first 15 min of fluid resuscitation. This effect diminished during prolonged fluid resuscitation. Furthermore, NEEP impaired lung mechanics without deterioration of gas exchange parameters. It could be proposed that the application of NEEP during severe hypovolemia could be useful during initial fluid resuscitation. However, this finding needs to be confirmed in a clinical setting.

## Funding sources

This research was planned and conducted in collaboration with the Department of Vascular Surgery, which received research funding from the hemostatic agent manufacturer Speed Care Mineral GmbH, which paid for the animals and consumables. The funder was not involved in the execution of the experiment.

## CRediT authorship contribution statement

**Julia Abram:** Writing – original draft, Project administration, Methodology, Investigation, Data curation, Conceptualization. **Patrick Spraider:** Writing – review & editing, Validation, Methodology, Investigation, Data curation, Conceptualization. **Julian Wagner:** Writing – review & editing, Investigation, Data curation. **Manuela Ranalter:** Writing – review & editing, Investigation, Data curation. **Alexandra Gratl:** Project administration, Methodology, Investigation, Data curation. **Daniela Lobenwein:** Writing – review & editing, Investigation, Data curation. **Sabine Wipper:** Supervision, Resources. **Gabriel Putzer:** Supervision, Resources. **Tobias Hell:** Visualization, Formal analysis, Data curation. **Pia Tscholl:** Writing – review & editing, Visualization, Formal analysis, Data curation. **Judith Martini:** Writing – review & editing, Supervision, Conceptualization.

## Ethics approval

This study was approved by the Institutional Animal Care and Use Committee and the national Ministry of Science, Research and Economy (Protocol No.: BMBWF-V/3b 2021–0.150.421) and was executed at the experimental research facility of the Department of Anesthesia and Intensive Care Medicine in collaboration with the Department of Vascular Surgery. This ventilation study was conducted as a supplement to a study of two different hemostatic agents for the treatment of vascular injuries, leading to a reduction of experimental animals by the 3R principle of animal research. It was performed in accordance with EU regulations for animal experimentation (EU-Directive 2010/63 of the European Parliament and the European Council) and reporting follows the ARRIVE 2.0 guidelines.^5^

## Declaration of competing interest

The authors declare that they have no known competing financial interests or personal relationships that could have appeared to influence the work reported in this paper.
